# Compositional analysis of ruminal bacteria from ewes selected for somatic cell score and milk persistency

**DOI:** 10.1371/journal.pone.0254874

**Published:** 2021-07-26

**Authors:** Guillermo Martinez Boggio, Annabelle Meynadier, Pepus Daunis-i-Estadella, Christel Marie-Etancelin

**Affiliations:** 1 GenPhySE, INRAE, INPT, ENVT, Université de Toulouse, Castanet-Tolosan, France; 2 Department of Computer Science, Applied Mathematics and Statistics, University of Girona, Girona, Spain; Tokat Gaziosmanpasa Universitesi, TURKEY

## Abstract

Ruminants are dependent on their rumen microbiota to obtain energy from plants. The composition of the microbiome was well-known to be associated with health status, and production traits, but published results are difficult to reproduce due to large sources of variation. The objectives of this study were to evaluate the associations of ruminal microbiota and its association with genetic lines selected by somatic cell score (SCS) or milk persistency (PERS), as well as milk production, somatic cell score, fat and protein contents, and fatty acids and proteins of milk, using the principles of compositional data. A large sample of 700 Lacaune dairy ewes from INRAE La Fage feeding the same diet and belonging to two divergent genetic lines selected for SCS or PERS was used. The ruminal bacterial metagenome was sequenced using the 16S rRNA gene, resulting in 2,059 operational taxonomic units affiliated with 112 genera. The abundance data were centred log-transformed after the replacement of zeros with the geometric Bayesian method. Discriminant analysis of the SCS showed differences between SCS+ and SCS- ewes, while for PERS no difference was obtained. Milk traits as fat content, protein content, saturated fatty acids and caseins of milk were negatively associated with *Prevotella* (R = [-0.08;-0.16]), *Suttonella* (R = [-0.09;-0.16]) and *Ruminococcus* (R = [-0.08;-0.16]), and positively associated with *Lachnospiraceae* (R = [0.09;0.16]) and *Christensenellaceae* (R = [0.09;0.16]). Our findings provide an understanding of the application of compositional data to microbiome analysis, and the potential association of *Prevotella*, *Suttonella*, *Ruminococcaceae* and *Lachnospiraceae* with milk production traits such as milk fatty acids and proteins in dairy sheep.

## Introduction

Ruminants are able to obtain energy from plant fibre to produce foods for human consumption. This is achieved through rumen symbiosis with colonizing microorganisms, such as bacteria, protozoa and fungi. Bacteria are the most abundant microorganisms in the rumen and make the greatest contribution to the digestion and conversion of feeds to short-chain fatty acids, microbial proteins and vitamins [1]. Associations of the ruminal microbiota with sire breed [2] and with different traits, such as feed efficiency [3], methane yield [4–6], and milk composition [7–9], have been reported, mainly in cows. However, in sheep only a few studies reported changes in the rumen bacteria with different diets [10–12], but no associations with milk production traits in dairy ewes. Research on cows considered a few animals with a maximum sample size of 16 [7–9] and used phenotypic differences, not genetic selection.

The main problem in published studies concerning the association between the microbiome and production traits is reproducibility. In the general workflow of microbiome analysis, the sources of variation, from sampling to statistical analysis, are almost infinite [13]. High-throughput sequencing technologies have made an important contribution to the knowledge of ruminal microbiome diversity. However, technologies with a limited number of sequencing reads obtained per sample, such as metabarcoding of the 16S rRNA gene, place a constraint on microbial data. Thus, the observed read counts is a fixed-size random sample of the relative abundance of the operational taxonomic units (OTUs) in the ecosystem. Moreover, the counts obtained are not related to the absolute value of the OTU, but to the probability of counting the OTU [14].

This kind of data is referred to as compositional, and a statistical approach adapted to this data must be applied. The term compositional data [15, 16] is used to describe a data set in which the parts of each sample have an arbitrary or noninformative sum, such as 100 for percentages. As result, the data contain only information about the relationships between the different parts of the composition. Three principles should be fulfilled in any statistical analysis of compositions: scale invariance, permutation invariance and sub-compositional coherence [16]. To meet one of the most important principles, scale invariance, it was proposed to work with the log-ratio, whose invariant form is called the log-contrast [15]. Compositional data are represented in a non-Euclidean space called a simplex. The log-ratio transformations proposed by Aitchison [15] and Egozcue et al. [17] allow observations to be represented in Euclidean space, on which most association analyses are statistically based. Centred log-ratio (CLR) and isometric log-ratio (ILR) transformations are the most widely used types of log-ratio transformations; both are isometric and allow correct operation in Euclidean space. However, only the ILR is orthonormal, generating a complete set of independent transformed variables on an orthonormal basis (as a coordinate system). Thus, the ILR works with balances [17], while the CLR works with OTU abundance, which allows a simple interpretation of the results.

Zero values are slightly more problematic in compositional data analysis than in standard multivariate statistical analysis because it is not possible to work with log-ratios if we have zero values in the data set. Microbiome metabarcoding data represent the probabilities of counts per OTU through a random sampling process [18], so some values in the data are true zero values due to true absence in the ecological environment, while others could arise randomly because of the constraint generated by high-throughput sequencing technologies. In the literature, different ways of correcting these zero values are applied, from the use of arbitrary corrections such as adding an offset of 1 to all values in the data set to the use of Bayesian models [18, 19].

Another procedure that must be considered when working with microbiome data, which contributes to the reproducibility of the results, is adjusting the data according to the different sources of experimental variation mentioned above. In the literature, these effects are known as batch effects, and they can include technical factors such as sample collection and storage, sample processing, and DNA sequencing; biological factors such as animal breed, health status and environmental effects; and computational factors such as bioinformatic pipelines and the statistical analysis used [20].

Thus, to obtain robust and reproducible results when working with microbiome data, it is crucial to use a compositional data approach, as stated by Gloor et al. [14] (*“Microbiome datasets are compositional*: *and this is not optional”*), and to adjust the data according to the principal sources of experimental variation.

The main purposes of this study are to present a conceptual framework for the compositional data approach applied to metabarcoding data in a discriminant analysis of divergent genetic lines of sheep selected on the basis of either somatic cell score (SCS) or milk persistency (PERS) and to link ruminal bacteria with milk production and milk quality traits.

## Materials and methods

### Animal handling and sampling

Data were obtained from the INRAE Experimental Unit of La Fage (UE 321 agreement A312031, Roquefort, France) between 2015 and 2019. The animals under study were adult Lacaune dairy ewes (weighing 77 kg on average) raised indoors and fed 93% meadow hay and silage plus 7% of concentrates (on dry matter basis). The genetic structure of the INRAE La Fage flock includes independent divergent genetic lines of Lacaune dairy ewes: two selected for milk SCS and the other two for PERS. Divergent selection based on estimated breeding values (EBVs) for milk SCS of sires of the whole Lacaune population and dams within the La Fage flock was initiated in 2003 [21]. Two groups of ewes with extreme EBVs were created according to the log-transformed somatic cell count (SCC): a high-SCS line, represented as SCS+, and a low-SCS line, represented as SCS-. This selection was demonstrated to produce ewes with susceptibility/resistance to natural clinical and sub-clinical mastitis [22]. Estimated breeding values of Lacaune sires were estimated relative to the whole Lacaune population based on the coefficient of variation of milk production on the testing day. Starting in 2009, extreme sires were mated to produce the PERS divergent lines in the La Fage flock. Two extreme groups of ewes were generated, one with high persistence (PERS+) and one with low persistence (PERS-) in the milk production curve.

Ruminal contents were sampled from each ewe using a vacuum pump and a medical gastric tube, that allows a qualitative representation of the rumen microbial community in a large number of animals [23]. To avoid dilution of samples by feed or water, the animals did not have access to feed and water 10 hours and 2 hours prior to sampling, respectively. Immobilization was performed with a special cage adapted for ewes, sampling was performed by competent staff, and the gastric tube was thoroughly rinsed with clean water between animal sampling to minimize cross-contamination. The rumen samples were directly aliquoted, frozen and stored at -80°C. This protocol received approval from the Ministere de l’Enseignement Superieur de la Recherche et de l’Innovation–Animal ethics committee with the following approval number: APAFIS#6292–2016080214271984 v8.

The experimental data consisted of 700 ruminal samples, including 94 from SCS+ ewes, 204 from SCS- ewes, 200 from PERS+ ewes and 202 from PERS- ewes. The genetic difference within the SCS and PERS lines was obtained by estimating index differences between SCS+/SCS- and PERS+/PERS- expressed in standard deviations of the indexes estimated for the whole Lacaune dairy population.

### 16S rRNA gene amplicon sequencing

Total DNA from 80 μL of ruminal sample was extracted and purified using the QIAamp DNA Stool Mini Kit (Qiagen Ltd, West Sussex, UK) according to the manufacturer’s instructions, with a previous bead-beating step in a FastPrep instrument (MP Biomedicals, Illkirch, France). The 16S rRNA V3-V4 regions of the extracted DNA strands were amplified (first PCR: 30 cycles) from purified genomic DNA with the primers F343 (5′–CTTTCCCTACACGACGCTCTTCCGATCTACGGRAGGCAGCAG–3′; [24]) and reverse R784 (5′–GGAGTTCAGACGTGTGCTCTTCCGATCTTACCAGGGTATCTAATCCT–3′; [25]). As Illumina MiSeq technology enables 250-bp reads, the ends of each read are overlapped and can be stitched together to generate full-length reads of the entire V3 and V4 regions in a single run. Single multiplexing was performed using a 6-bp index, which was added to R784 during a second PCR with 12 cycles using forward primer (AATGATACGGCGACCACCGAGATCTACACTCTTTCCCTACACGAC) and reverse primer (CAAGCAGAAGACGGCATACGAGATGTGACTGGAGTTCAGACGTGT). The PCR products were purified and loaded onto an Illumina MiSeq cartridge (Illumina, San Diego, CA, USA) at the Genomic and Transcriptomic Platform (INRAE, Toulouse, France) according to the manufacturer’s instructions. This process was repeated each year between 2015 and 2019, but in the first three years, the sequencing process was carried out at different times, so the samples were not sequenced in the same batch.

The sequences of the 700 samples were processed using the FROGS pipeline [26] by following the FROGS workflow operational procedure: (i) read demultiplexing, i.e., assigning each paired-end read to its sample on the basis of the previously integrated index; (ii) read pre-processing, i.e., removing sequences presenting a primer mismatch, displaying an unexpected length (>300 or <500 bp) or with at least one ambiguous base; (iii) chimaera removal; (iv) sequence clustering with denoising and a defined difference of d = 1 between sequences in each aggregation step of clustering; (v) cluster filtering, i.e., removing clusters with abundances <0.005% of the total sequences [27]; and (vi) taxonomy assignment to OTUs using SILVA database (version 138). OTU number refers to the identification of the OTU in the abundance table.

### Abundance data

The abundance table and taxonomy files were imported into R (v4.0.2). Zeros were imputed under the assumption that the probability of occurrence of the OTUs in the multinomial experiment was not zero. Therefore, the geometric Bayesian-multiplicative (GBM) method [19] was used, where the zero values were replaced in each sample by the posterior probability obtained from the Bayesian model, which considered all the available data, and weighted by the geometric mean. To maintain the ratios between all the abundance values, the non-zero values were multiplied by a value generated as a function of the posterior probability and the geometric mean. The GBM method was performed with the following formula, through the cmultRepl function of the zCompositions package [28] in R (v4.0.2):

$$r_{ij}=\{\begin{array}{c} \frac{{\hat{m}}_{ij}}{g_{i}\cdot n_{i}+1},\;if\;x_{ij}=0,\\ x_{ij}\cdot \left(1-\frac{\Sigma_{k|x_{ik}=0}{\hat{m}}_{ik}}{g_{i}\cdot n_{i}+1}\right),\;if\;x_{ij}>0. \end{array}$$ (1)

where *r*_*ij*_ is a vector of replacement abundance values defined as *r*_*ij*_ = (*r*_*i*1_,…,*r*_*i*2059_), ${\hat{m}}_{ij}$ is the prior probability estimate for each category j, *g*_*i*_ is the geometric mean of the i-th row, and *n*_*i*_ is the number of samples.

The abundance table with no zero values was CLR transformed with the following formula through the compositions package [29] in R (v4.0.2):

$$clr(x)=[\log\left(\frac{x_{1}}{g(x)}\right),\log\left(\frac{x_{2}}{g(x)}\right),\ldots ,\log\left(\frac{x_{D}}{g(x)}\right)],$$ (2)

where *x* is a row vector with abundances for the OTUs in the sample (*x*_1_ = OTU 1, *x*_2_ = OTU 2, to *x*_*D*_ = OTU 2059), $g(x)=\sqrt[{D}]{x_{1}\cdot x_{2}\cdot \ldots \cdot x_{D}}$, is the geometric mean of *x*, and *D* is equal to 2,059.

The CLR-transformed bacterial abundances were adjusted with a unique general linear model, performed with the sasLM package in R (v4.0.2), and the fixed effects that were significant (P<0.05) for more than 10% of the OTUs were retained.

Finally, the model was:

y=μ+α*DIM+Nseq+Year+Run(Year)+Lact(Year)+Hour(Year)+e (3)

where *y* is the CLR-transformed traits of the OTUs, *μ* is the overall mean, *DIM* is the lactation stage (from 28 to 133 days in milk) included as a covariable, *Nseq* is the number of sequences per sample as a fixed effect (7 levels from <5,000 to >30,000 sequences), *Year* is the year fixed effect (6 levels), *Run*(*Year*) is the fixed effect of run within year (5 levels), *Lact*(*Year*) is the fixed effect of lactation number within year (3 levels), *Hour*(*Year*) is the fixed effect of the hour of sampling in the morning/afternoon within year (8 levels), and *e* is the residual random effect.

The genetic line effect represented by differences among SCS+, SCS-, PERS+ and PERS- was not considered in the model since it was used as a discriminating factor in the multivariate discriminant analysis.

### Phenotypic data

Daily recordings of milk production, milk somatic cell count (SCC) quantified with a Fossomatic cell counter (Foss, Nanterre, France), and milk fat and protein contents (FC and PC) were performed as part of the official milk recording of the flock. Ruminal samplings were performed between 0 and 3 days after the milking recordings were made in the morning and afternoon milkings. Two samples per animal were sent for analysis at the Interprofessional Milk Analysis Laboratory (Agrolab’s Aurillac, France). Milk FC and PC were analysed with mid-infrared (MIR) techniques with a Milko-Scan^TM^ FT6000 instrument (Foss, Nanterre, France). The daily milk production traits studied were daily milk yield (MP), daily FC and PC (as weighted averages), and daily SCS [SCS = 3 + log_2_(SCC/100,000)].

Moreover, for these official milk recordings (with the exception of those made in 2016), the MIR spectra were recovered in order to predict the fine profile of milk proteins and fatty acids. Fresh milk samples were analysed using MIR spectrometry [30]. The spectral data of the individual milk samples were obtained on a Milko-Scan^TM^ FT6000 instrument (Foss, Nanterre, France). The proteins included in the analysis were the 4 caseins (CNs) α_s1_-CN, α_s2_-CN, β-CN and κ-CN and the 2 soluble proteins α-lactalbumin and β-lactoglobulin [31]. The saturated fatty acids (SFAs), unsaturated fatty acids (UFAs) and polyunsaturated fatty acids (PUFAs) included in the analysis were only the FAs used in ewe milk predictions [30], such as butyric acid (C4:0), caproic acid (C6:0), caprylic acid (C8:0), capric acid (C10:0), lauric acid (C12:0), palmitic acid (C16:0), oleic acid (*cis-9* C18:1), conjugated linoleic acid (*cis-9 trans-11* C18:2) and α-linoleic acid (C18:3*n-3*). Milk proteins and fatty acids are expressed in g per 100 ml.

The daily FC and PC, milk proteins and milk FAs were CLR transformed to account for their compositional nature, and all traits were adjusted using the sasLM package in R (v4.0.2) according to:

y=μ+α*DIM+Year+Lact(Year)+e (4)

where *y* is the milk production traits; *μ* is the overall mean; *DIM* is the lactation stage (from 28 to 133 days in milk) included as a covariable; *Year* is the fixed effect of year (6 levels); *Lact*(*Year*) is the fixed effect of lactation number within year (3 levels); and *e* is the residual random effect.

### Multivariate analysis

The multivariate analysis was performed with the residuals obtained from Eq (3) for bacterial abundances and Eq (4) for milk traits.

Two discriminant analyses were performed on OTU abundances to discriminate the divergent lines (for SCS and PERS separately), using sparse partial least-squares discriminant analysis (sPLS-DA). The number of components selected was based on principal component analysis, from which the sum of components explained at least 60% of the variation. The number of variables was selected using the CLR-lasso penalty method considering the optimal number as a function of the lambda value after 25-fold cross-validation. The loading values indicate the weight of a subset of OTUs whose linear combination maximizes the differences between genetic lines.

Regression analyses of the relationships of ruminal bacteria with milk production traits and MIR-predicted traits performed on all divergent lines together, using sparse partial least-squares (sPLS) analysis. A single sPLS analysis was carried out for milk production traits and fine milk FA and protein compositions predicted with MIR. The analysis included 561 ewes with information for all traits. As previously described, principal components analysis and the CLR-lasso penalty method were used to define the numbers of components and variables for the sPLS model. The multivariate analysis were implemented using mixOmics package [32] in R (V4.0.2). A Pearson correlation matrix was calculated with only the OTUs selected according to the first principal component (PC1) and second principal component (PC2) of the corresponding sPLS analysis. Statistical significance was declared at a P value <0.05. Then, clustering of OTUs and traits was performed with the heatmaply function in R (v4.0.2).

The classification reliability corresponding to the discriminant analysis model was assessed as a function of the maximum prediction distance between the overall misclassification error rate and balanced error rate (BER) after fivefold cross-validation repeated 10 times. BER was calculated as 1 –balanced accuracy.

## Results

As a result of the bioinformatics analysis, 9,536,442 sequences were retained (63% of the initial total DNA sequences). The abundance table included 2,059 affiliated OTUs, represented by 751 to 168,617 sequences (mean of 1,761 DNA sequences). Rare OTUs represented by fewer than 2,034 sequences across all samples were excluded from the analysis. Genera were defined as the finest taxonomic level due to an unknown species frequency of 94%.

**Fig 1:**
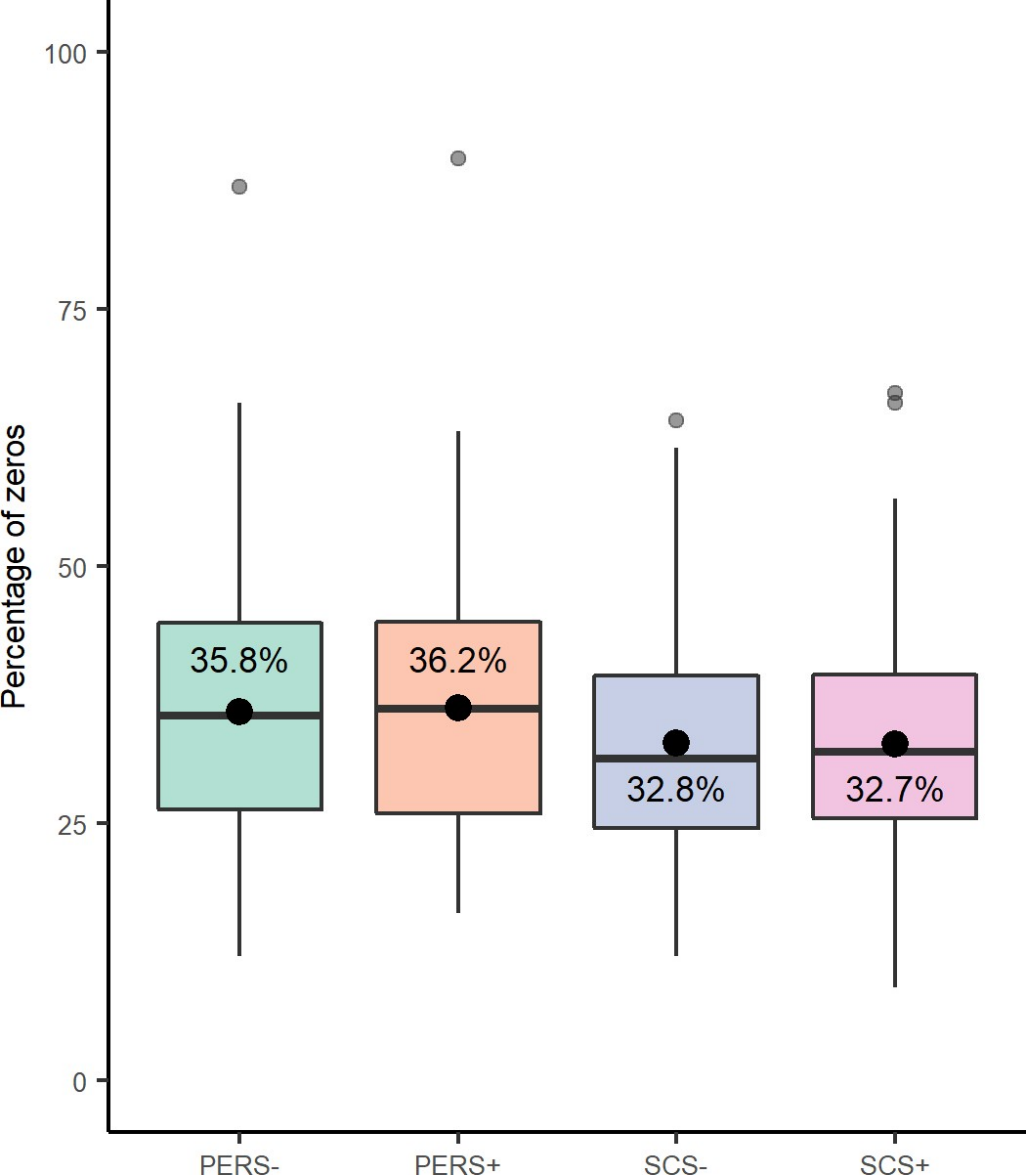
Percentage of zero values in data by genetic line. SCS+ and SCS- as somatic cell score lines susceptibility/resistance, and PERS+ and PERS- as milk persistency line high/low persistence.

Overall, the 2,059 OTUs from the 700 samples were attributed to 11 phyla and 112 genera. Expressed as a percentage of total sequences for all samples, the most representative phyla were *Bacteroidetes* (50.8%), *Firmicutes* (43.3%) and *Proteobacteria* (2.7%), and the most abundant genera were *Prevotella* (34%), *Lachnospiraceae NK3A20 group* (6.4%), *Ruminococcus* (5.8%), *Christensenellaceae R-7 group* (5.3%), *Oscillospiraceae NK4A214 group* (3.8%) and *Rikenellaceae RC9 gut* (3.6%). The percentage of zero values in whole abundance table is shown in Fig 1.

### Discriminant analysis of SCS and PERS lines

Divergent selection created large differences between the lines: 2.19 units of SCS EBVs (i.e., 3.6 genetic SD) created between the 94 SCS+ and 204 SCS- ewes and 5.52 units of milk CV EBVs (i.e., 2.1 genetic SD) created between the 200 PERS+ and 202 PERS- ewes.

**Fig 2:**
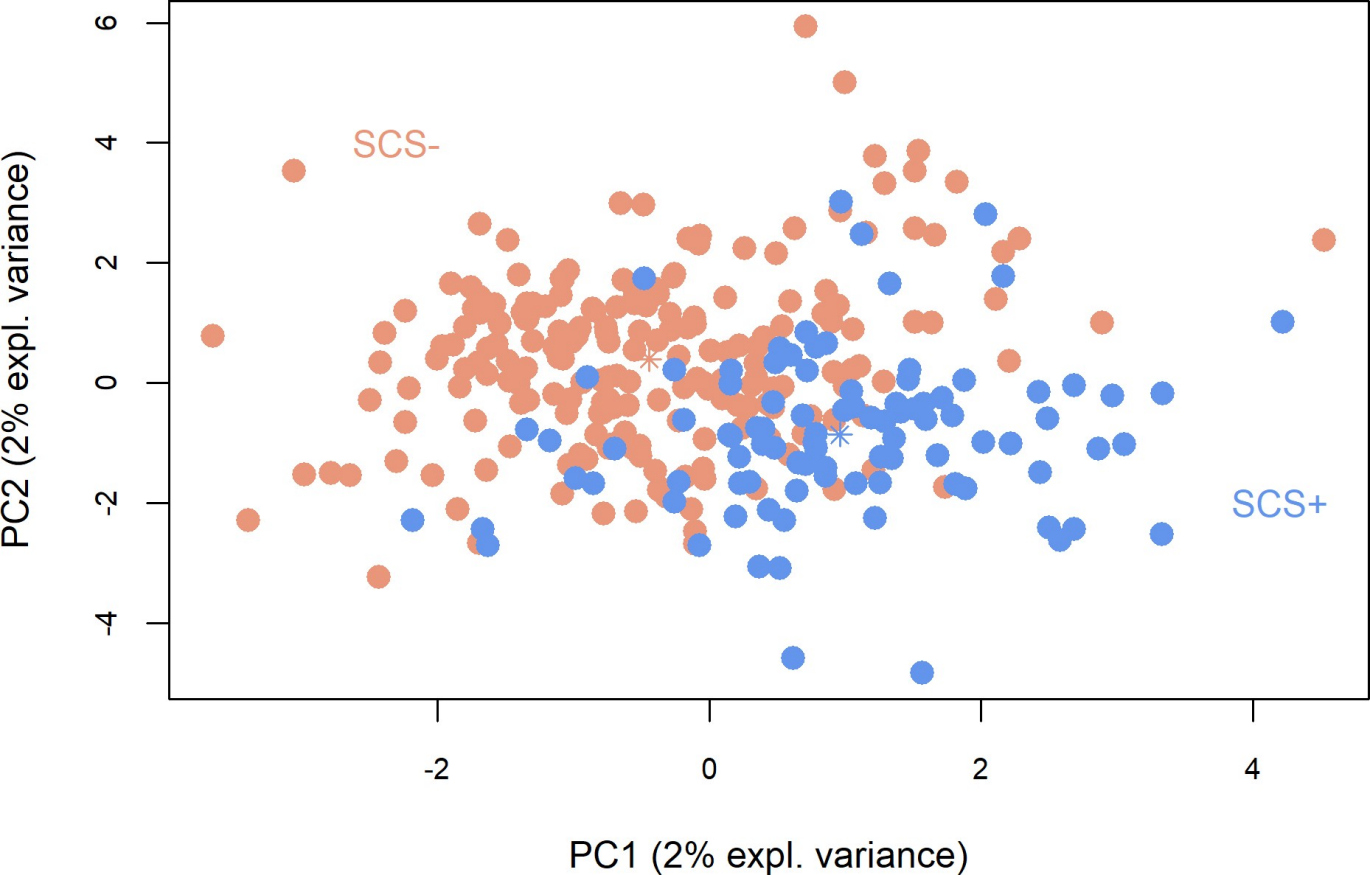
Sparse partial least squares discriminant analysis between divergent somatic cell score (SCS) lines. SCS+: ewes selected for high somatic cell score i.e. susceptible to mastitis; SCS-: ewes selected for low somatic cell score i.e. resistant to mastitis.

The discriminant model defined for SCS lines included 100 principal components (63% of variance explained) and 17 variables. The SCS+ and SCS- ewes could be discriminated on the basis of their ruminal bacteria (Fig 2). Table 1 includes the 34 OTUs most associated with the SCS lines in each of the first 2 principal components. Only two OTUs were removed from the abundance table because of zero values for all samples. The BER obtained from the model was 0.50, and the first two principal components explained 4% of the variance.

**Table 1:** Loading values per OTU with genus affiliation, associated genetic line and percentage of abundance, for the two first components from the somatic cell score (SCS) line sparse partial least squares discriminant analysis.

Affiliated *genus*	OTU	PC1	Line	Abundance (%)	Affiliated *genus*	OTU	PC2	Line	Abundance (%)
*Prevotella*	14	0.40	SCS+	0.80	*Prevotella*	1064	0.35	SCS-	0.01
*Ruminococcaceae/ unknown genus*	501	0.34	SCS+	0.04	*Prevotella*	1092	0.34	SCS-	0.05
*Prevotella*	819	0.27	SCS+	0.01	*Ruminococcus*	657	0.32	SCS-	0.02
*Prevotellaceae UCG-001*	467	0.25	SCS+	0.08	*Prevotella*	112	0.23	SCS+	0.13
*F082/unknown genus*	3286	0.13	SCS+	0.01	*Lachnospiraceae NK4A136 group*	2186	0.22	SCS-	0.01
*Lachnospiraceae AC2044 group*	230	0.08	SCS+	0.08	*Prevotellaceae YAB2003 group*	517	0.17	SCS-	0.10
*Prevotella*	1486	0.08	SCS+	0.01	*Anaeroplasma*	220	0.16	SCS-	0.06
*Lachnospiraceae NK3A20 group*	625	-0.06	SCS-	0.02	*Prevotella*	1145	0.11	SCS-	0.03
*Clostridia UCG-014/ unknown genus*	530	-0.09	SCS-	0.05	*Christensenellaceae R-7 group*	382	-0.02	SCS+	0.19
*Prevotella*	1145	-0.12	SCS-	0.03	*F082/unknown genus*	2208	-0.03	SCS+	0.01
*Moryella*	328	-0.15	SCS-	0.04	*Pseudoramibacter*	226	-0.08	SCS+	0.04
*Syntrophococcus*	1739	-0.19	SCS-	0.01	*Christensenellaceae R-7 group*	285	-0.19	SCS+	0.17
*Fibrobacter*	1640	-0.23	SCS-	0.02	*Family XIII UCG-001*	450	-0.24	SCS+	0.04
*F082/ unknown genus*	175	-0.26	SCS-	0.08	*Mogibacterium*	79	-0.26	SCS-	0.17
*Prevotella*	367	-0.33	SCS-	0.05	*Prevotella*	443	-0.29	SCS+	0.05
*Prevotella*	1842	-0.34	SCS-	0.01	*Lachnospiraceae NK3A20 group*	39	-0.31	SCS+	0.27
*Christensenellaceae R-7 group*	2009	-0.36	SCS-	0.01	*Oscillospiraceae/ unknown genus*	538	-0.36	SCS+	0.04

When the genus is unknown, the family affiliation is included before the backslash (/).

OTU, identification number of the operational taxonomic unit.

PC1, first principal component; PC2, second principal component.

Abundance (%), expressed as percentage of total sequences.

The *Prevotella* genus was well represented, with 11 OTUs associated with either the SCS+ or the SCS- ewes, through components 1 and 2 (Table 1). Only OTU1145 was associated with SCS- ewes for the two main components. The *Christensenellaceae R-7 group* genus appeared to be associated with SCS- ewes in PC1, but in PC2, OTU285 and OTU382 belonging to this same genus were associated with SCS+ ewes. The family *Lachnospiraceae* was well represented by *Lachnospiraceae NK3A20 group*, *Lachnospiraceae NK4A136 group* and *Lachnospiraceae AC2044 group*, which were associated with either SCS- or SCS+ ewes.

**Fig 3:**
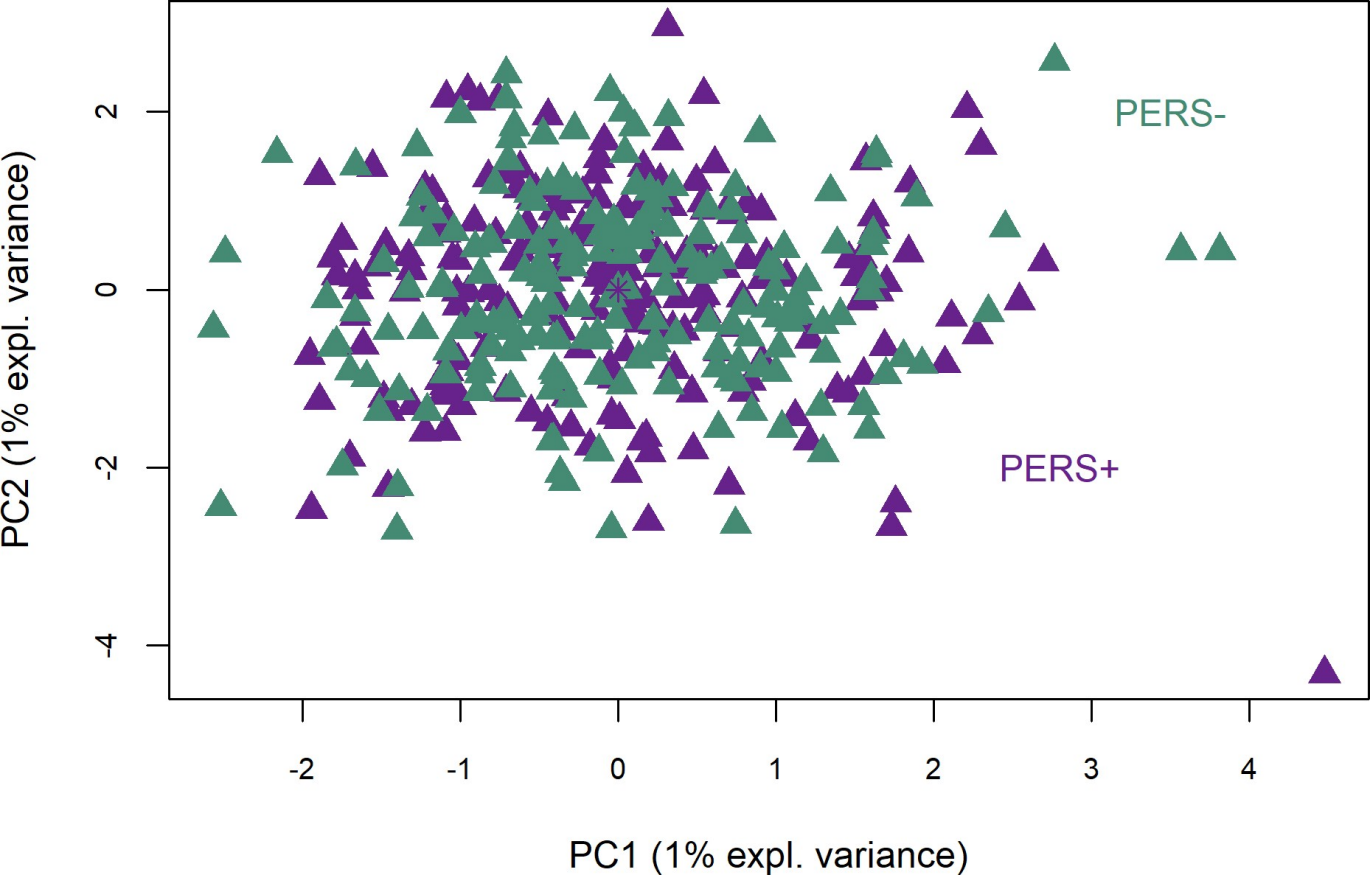
Sparse partial least squares discriminant analysis between divergent milk persistency (PERS) lines. PERS+: ewes selected for a high milk persistence; PERS-: ewes selected for a low milk persistence.

The discriminant model for PERS lines included 120 principal components (62% of variance explained) and 5 variables. The PERS+ and PERS- lines could not be discriminated according to their ruminal bacteria (Fig 3). Table 2 includes the 10 OTUs most associated with the PERS lines in each of the first 2 principal components. The BER obtained from the model was 0.71, and the first two principal components explained 2% of the variance. The *Prevotella* genus, represented by OTU1482 (PC1) and OTU1395 (PC2), was positively associated with PERS- ewes. In addition, the PERS- line was associated with *Oscillospiraceae NK4A214*, *Blautia* and an unknown genus (order *Clostridia UCG-014*) through component 1 and with *Streptococcus* through component 2. Thus, the genera *Ruminococcus* and *Oscillospiraceae NK4A214* were associated with PERS+ ewes.

**Table 2:** Loading values per OTU with genus affiliation, associated genetic line and percentage of abundance, for the two first components from the milk persistency (PERS) line sparse partial least squares discriminant analysis.

Affiliated *genus*	OTU	PC1	Line	Abundance (%)	Affiliated *genus*	OTU	PC2	Line	Abundance (%)
*Prevotella*	1428	0.79	PERS-	0.01	*[Eubacterium] coprostanoligenes group/ unknown genus*	1501	0.39	PERS+	0.01
*Clostridia UCG-014/ unknown genus*	411	-0.22	PERS-	0.04	*Prevotella*	1395	0.11	PERS-	0.01
*Blautia*	216	-0.29	PERS-	0.07	*Streptococcus*	634	-0.15	PERS-	0.04
*Oscillospiraceae NK4A214*	292	-0.34	PERS-	0.07	*Anaerovoracaceae/ unknown genus*	1131	-0.58	PERS+	0.02
*Ruminococcus*	537	-0.36	PERS+	0.05	*Oscillospiraceae NK4A214*	823	-0.69	PERS+	0.02

When the genus is unknown, the family affiliation is included before the backslash (/).

OTU, identification number of the operational taxonomic unit.

PC1, first principal component; PC2, second principal component.

Abundance (%), expressed as percentage of total sequence.

### Regression analysis between ruminal bacterial abundance and milk traits

**Fig 4:**
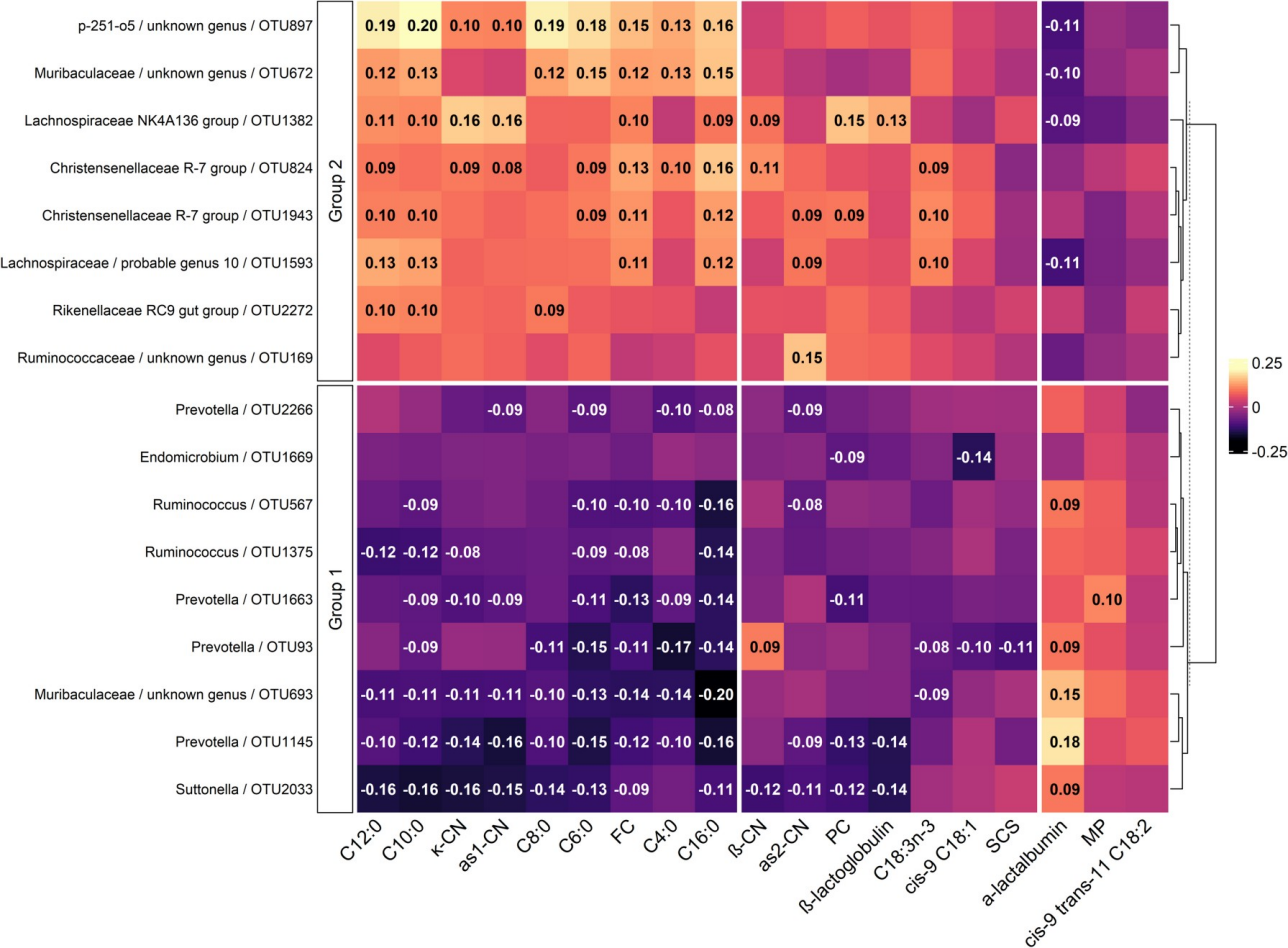
A correlation matrix heatmap between bacterial taxa and milk traits. OTUs selected by the 2 first components of the sparse least squares analysis; daily milk production (MP), somatic cell score (SCS), daily milk protein contents (PC), daily milk fat content (FC), milk fatty acids (butyric acid (C4:0), caproic acid (C6:0), caprylic acid (C8:0), capric acid (C10:0), lauric acid (C12:0), palmitic acid (C16:0), oleic acid (*cis-9* C18:1), conjugated linoleic acid (*cis-9 trans-11* C18:2) and α-linoleic acid (C18:3*n-3*), expressed as % of total fatty acids) and milk proteins, as casein (CN) (α_s1_-CN, α_s2_-CN, β-CN, κ-CN, expressed as % of total proteins), and soluble proteins (α-lactalbumin and β-lactoglobulin, expressed as % of total proteins).

The sPLS regression model included 150 components and 9 variables. Fig 4 shows only the 17 most representative OTUs from PC1 and PC2 (OTU1593 was representative for both components and all traits).

Daily milk production and SCS were each correlated with one OTU of the genus *Prevotella* (Fig 4). Milk FC and PC had similar correlations with 5 common OTUs: they were negatively correlated with 2 *Prevotella* OTUs (R = [-0.11;-0.13], P< 0.01) and with *Suttonella* (R = [-0.09;-0.12], P< [0.05;0.01]) and positively correlated with *Lachnospiraceae NK4A136 group* (R = [0.10;0.15], P< [0.05;0.01]) and *Christensenellaceae R-7 group* (R = [0.09;0.11], P< [0.05;0.01]). Moreover, PC was specifically correlated with *Endomicrobium*, while FC had numerous correlations, such as positive correlations with *Lachnospiraceae probable genus 10* and *Christensenellaceae R-7 group*, negative correlations with 2 *Ruminococcus* OTUs and variable correlations with 2 OTUs of the *Muribaculaceae* family (R = [0.12;-0.14], P< 0.01).

α_s1_-CN, κ-CN and β-lactoglobulin were positively correlated with *Lachnospiraceae NK4A136 group* (R = [0.13;0.16], P< 0.01) and negatively correlated with *Prevotella* and *Suttonella* (R = [-0.14;-0.16], P< 0.01). To a lesser extent, *Christensenellaceae R-7 group* and the family *Muribaculaceae* showed positive and negative correlations with α_s1_-CN and κ-CN, respectively (Fig 4). α_s2_-CN and β-CN exhibited the same trend as the other caseins but with weaker correlations: negative correlations with *Suttonella* and *Prevotella* and positive correlations with *Lachnospiraceae NK4A136 group*. α-Lactalbumin was clearly different from the other protein since it was positively correlated with *Prevotella* and with an unknown genus of the family *Muribaculaceae* (R = [0.15;0.18], P< 0.001), while the families *Lachnospiraceae* and p-251-o5 showed negative correlations with this protein (R = -0.11, P< 0.01).

The strongest correlations were observed with SFAs, which were negatively correlated with all 4 *Prevotella* OTUs selected by sPLS analysis and with *Suttonella*, particularly for C10:0 and C12:0 (R = -0.16, P< 0.001). Some genera of the phylum *Firmicutes* were correlated with SFAs. For example, 2 OTUs belonging to *Christensenellaceae R-7 group* were positively correlated with SFAs, and 2 OTUs belonging to *Ruminococcus* were negatively correlated with SFAs. An unknown genus of the *Muribaculaceae* and the p-251-o5 family showed the maximum correlations of 0.20 (P< 0.001) with C10:0.

Compared to SFAs, MUFAs had fewer significant correlations with OTUs. As presented in Fig 4, the MUFA *cis-9* C18:1 was negatively associated with *Endomicrobium* and *Prevotella*, while the PUFA C18:3*n-3* was positively associated with *Christensenellaceae R-7 group* and *Probable genus 10* and negatively associated with *Prevotella* and an unknown genus of *Muribaculaceae*. Finally, *cis-9 trans-11 C18*:*2* was not correlated with any of the 17 OTUs selected by sPLS analysis.

## Discussion

*Bacteroidetes*, *Firmicutes* and *Proteobacteria* were the most dominant phyla in the rumen of dairy ewes. The same phyla were reported by other authors studying sheep [11, 33] and dairy cows [7–9, 34–37] with different rumen sampling methods and statistical analysis.

The analysis of microbiome abundance data with the commonly applied methodology [7–9, 34, 37], i.e., data treatment with a normalization process, such as rarefaction, and using nonmetric distances (i.e., Bray Curtis), provides results that seem satisfactory, irrespective of the compositional nature of the data. However, statistical knowledge since Pearson [38] has shown that processing such data without considering them as compositional could lead to spurious correlations. More recently, Gloor et al. [14] pointed out that the use of traditional methods to analyse data without considering their compositional nature can lead to “misleading and unpredictable” results [13, 14, 16].

Thus, this work aimed to apply compositional data analysis to the rumen bacterial metagenome obtained by metabarcoding and to correct for technical and zootechnical effects in order to obtain robust and reproducible results. The compositional workflow of the study consisted of the following steps:

1. Zero values were corrected with the GBM method [19]. Theoretically, this method is appropriate since it generates a minor distortion in the ratios between OTU abundances, based on the correction of zero values and the multiplication of non-zero values. In addition, the GBM method considers the multivariate nature of microbiome data through a Bayesian model, where new values are generated on the basis of the posterior probabilities of zero values in the raw data.
2. OTU abundance was CLR transformed. This transformation allows a simple interpretation of the biological results, since each OTU in each sample is compared with the geometric mean of the sample. The limit of CLR transformation is that OTUs remain dependent because of the use of the geometric mean. Therefore, CLR transformation partially solves the problem identified by Pearson in 1897 [38]. However, the statistically correct alternative to CLR transformation proposed by Egozcue et al. [17], i.e., the ILR transformation, does not allow easy interpretation of the results [39]. Indeed, ILR transformation works with balances (linear combinations of OTUs) to achieve total independence among the OTUs, and it is currently not possible to back-transform the results after multivariate analyses. Further work is needed in this sense.
3. The microbiome and phenotypic data were adjusted through linear models. These models must include in their definition batch effects [20] which are any unwanted source of variation representing biological and technical effects. When the effects are balanced in the experiment, linear models are an interesting method to correct for batch effects [20]. From these models, the residual values (variation not explained by the included effects), which are ultimately the input values for multivariate analyses, are obtained. However, the consequence of using residual values for sPLS-DA and sPLS analyses is that the remaining variation in the residuals exploited by these regression models is reduced, as shown below.

In this way, we considered not only the nature of the available microbiome data to work in the appropriate geometric space (Euclidean) but also the residuals to allow a more correct analysis of the effect under study, i.e., the genetic lines based on SCS and PERS.

### Discriminant analysis

Discriminant analysis for both the SCS and PERS lines showed low explained variance (Figs 2 and 3) for the first two principal components. Using residuals leads to a smaller variance of the values and therefore affects the variance explained by the discriminant effect for the first components. As a result, it is necessary to include a large number of components in the analysis. Variable selection was performed through the CLR-lasso method and allowed some OTUs with low abundance that carried irrelevant information to be excluded.

Since all ewes were Lacaune breed receiving the same diet and batch effects (except that of line) were corrected for by the linear model, the remaining variation in the rumen bacteria may be explained by the genetic lines. In spite of this, the variance among the genetic lines was not explained by the composition of the host animal’s microbiome for PERS, and only slightly for SCS. This is demonstrated by the BER obtained for the sPLS-DA analyses of 0.50 for SCS and 0.71 for PERS. Nevertheless, Fig 2 shows a slight difference between the SCS+ and SCS- lines, despite only 4% of the total variance being explained (for both components). Some OTUs assigned to *Prevotella*, *Christensenellaceae R-7 group* and unknown genus of the family *Ruminococcaceae* were the main discriminants for the first component (Table 1). Zhong et al. [36] did not report differences in these three genera between the rumens of cows with phenotypically high and low SCCs; in this comparison, the authors noticed only enrichment of *Proteobacteria* (especially an unclassified *Succinivibrionaceae*) in the ruminal microbiota of cows with low SCCs. In our study, these OTUs were not significantly different according to SCS. Therefore, the hypothesis of a link between selection on SCS and modifications in the rumen microbial population was not rejected, but its validity remains unclear in terms of the bacteria involved. The PERS line analysis revealed a complete absence of differences between PERS+ and PERS- ewes, as shown in Fig 3. However, three OTUs presented loading values greater than 0.5 (Table 2) along PC1 and PC2, and they belonged to *Prevotella*, *Oscillospiraceae NK4A214* and an unknown genus of *Anaerovoracaceae*. Nevertheless, there was no hypothesis of a correlated response of ruminal microbiome abundance to PERS selection.

The results for both divergent lines suggest that genetic selection for zootechnical traits, such as udder health and milk production curves, did not modify the abundance of rumen bacteria and therefore the animals’ ability to digest their feed.

### Links between ruminal bacteria and milk traits

Daily milk production was positively associated with a *Prevotella* OTU, similar to the results reported by Huang et al. [40]. This genus is known to have major metabolic activity in the production of propionate [41], which is the main precursor for gluconeogenesis in the liver [1] leading to lactose production. Some authors [9, 35] reported that some genera of the *Lachnospiraceae* family were positively correlated with daily milk yield, while we found that two OTUs affiliated with this family were weakly but negatively associated with daily milk production, as reported by Huang et al. [40]. The results obtained in dairy cows can be considered as references for dairy sheep, since as shown [42] the differences in terms of rumen microbiota among species are smaller when the diet is based on a mixture of forage and concentrates.

The SCS was correlated with a *Prevotella* OTU, but a possible association between this genus and the SCC in milk has not been reported, and these results are in line with the difficulty of differentiating the genetic lines selected for SCS. As expressed by Zhong et al. [36], the bacterial communities in the rumen are stable in animals with different SCCs, and this is probably true of ewes, where mastitis is overwhelmingly sub-clinical. However, the main hypothesis is a link between the intestinal microbiota and intramammary infection (i.e., clinical mastitis) [43].

Concerning milk composition, we identified two groups of OTUs (Fig 4): group 1, with negatively linked OTUs belonging to *Prevotella*, *Suttonella*, *Ruminococcus* and *Endomicrobium*, and group 2, with positively linked OTUs belonging *Lachnospiraceae NKA136*, *probable genus 10*, *Rikenellaceae RC9*, *Ruminococcaceae*, *Christensenellaceae* and *p-251-o5*. Muribaculaceae was represented by one OTU in the two groups, and for α-lactalbumin and daily milk production, the relation was reversed. Group 1 was represented mostly by propionic acid and proteolytic bacteria such as *Prevotella* [41], *Suttonella* [44] and some *Ruminococcus* [45], characterized by increasing milk production with a possible dilution of milk components. In contrast, group 2, with mostly butyric and acetic acid-producing bacteria such as *Lachnospiraceae* [46], had less proteolytic activity [47, 48], leading to the opposite effect for the concentration of milk components. These results are in accordance with other studies in dairy cows that also found *Prevotella*ceae family negatively correlated with milk fat, and *Lachnospiraceae* positively correlated with milk fat and protein contents [8, 37, 49].

In sheep [33] as well as in cows [50] a close relationship between the rumen microbiota composition and short-chain fatty acids in rumen was reported that could influence the synthesis of milk components. Therefore, the most likely hypothesis is that bacteria of group 2, through butyric and acetic acid, promote the production of short- and medium-chain SFAs.

In conclusion, this study applying the compositional data approach to a significant sample size of Lacaune dairy ewes revealed that rumen bacteria belonging to *Prevotella*, *Suttonella*, *Ruminococcaceae* and *Lachnospiraceae* are associated with milk production traits such as milk fatty acids and proteins. However, despite the large genetic differences between lines, ruminal bacteria were able to only weakly discriminate between SCS lines and unable to discriminate between PERS lines. Although dilution of the ruminal samples by saliva could be expected, correction of the rumen microbiota for the number of sequences per sample could have reduced this effect.

Since some abundant OTUs were correlated with milk composition traits, it would be interesting to further investigate the mechanism by which rumen bacterial metabolites affect milk composition traits in order to understand the relationships detected in this work.
